# Depressive symptoms in LGBT and heterossexual medical students: a comparative cross-sectional study

**DOI:** 10.1192/j.eurpsy.2026.11388

**Published:** 2026-07-31

**Authors:** A. F. C. Ximenes, L. V. D. Araujo, P. F. B. de Araújo, A. B. D. C. Alves, L. C. Carneiro, M. I. O. de Morais, F. O. Barbosa, B. D. A. A. L. T. de Melo, E. D. F. Correia, V. L. de-Melo-Neto, D. B. B. Ferreira

**Affiliations:** 1 Psychiatry, Universidade Federal de Alagoas; 2 Psychiatry, Universidade de Ciências da Saúde de Alagoas, Maceió, Brazil

## Abstract

**Introduction:**

Depression is one of the most prevalent psychiatric conditions among medical students, often driven by high academic demands, long study hours, and emotional burden. International studies suggest that LGBT students are at heightened risk, consistent with minority stress theory, which links stigma to worse mental health outcomes. However, results across settings are inconsistent, with some evidence pointing to resilience and protective community factors. In Brazil, comparative data on depressive symptoms in LGBT and heterosexual medical students are limited. Clarifying these patterns is crucial for tailoring mental health strategies to diverse student groups.

**Objectives:**

To compare depressive symptoms between LGBT and heterosexual medical students.

**Methods:**

Cross-sectional study in a medical school in Sergipe (Brazil). A total of 142 undergraduates (7 gay/lesbian, 3 bisexual, 132 heterosexual; minimum sample=84; eligibility ≥18 years; informed consent obtained) completed a sociodemographic questionnaire and the Beck Depression Inventory. Depression levels were classified as minimal, mild, moderate, or severe. Analyses included Fisher’s exact test and ANOVA.

**Results:**

Minimal depression predominated in both groups. Among LGBT students, 80% scored in the minimal range, while none reached severe depression. Only 10% presented mild to moderate symptoms. Conversely, 21% of heterosexual students reported mild to moderate depression. Mean BDI scores were similar (6.8 versus 7.1; p=0.857).

**Conclusions:**

Contrary to international literature, LGBT students did not show greater vulnerability to depressive symptoms. Heterosexual students presented more frequent mild to moderate symptoms, possibly related to gaps in social or family support. These results highlight the importance of addressing mental health broadly in medical education, considering protective and risk factors beyond sexual orientation. However, interpretations should be cautious, as the small LGBT subsample restricted statistical power and limited the generalizability of findings.

**Disclosure of Interest:**

None Declared

